# Sustainable Grape Antioxidant Dietary Fiber Preserves Proximal Colonic Homeostasis via Hsp27 and AMPK Signaling

**DOI:** 10.3390/ijms262110564

**Published:** 2025-10-30

**Authors:** Paula Ortega-Menéndez, Marina Hernández-Martín, Silvina Rosa Drago, Carlos Guillén, Jara Pérez-Jiménez, Dulcenombre Gómez-Garre, Luis Rivera, Verónica Azcutia, María Elvira López-Oliva

**Affiliations:** 1 Department of Physiology, Faculty of Pharmacy, Complutense University of Madrid, Plaza Ramón y Cajal s/n, 28040 Madrid, Spain; paulao03@ucm.es (P.O.-M.); marinh04@ucm.es (M.H.-M.); lrivera@ucm.es (L.R.); vazcutia@ucm.es (V.A.); 2 Department of Physiology, Faculty of Pharmacy, Complutense University of Madrid, Hospital Clínico San Carlos, IdISSC, c/Prof. Martín Lagos Street, 28040 Madrid, Spain; 3 Institute of Food Technology, National Scientific and Technical Research Council (CONICET), Faculty of Chemical Engineering, National University of the Littoral (FIQ-UNL), 1º de Mayo 3250, Santa Fe 3000, Argentina; sdrago@fiq.unl.edu.ar; 4 Department of Biochemistry, Faculty of Pharmacy, Complutense University of Madrid, IdISSC, Plaza Ramón y Cajal s/n, 28040 Madrid, Spain; cguillen@ucm.es; 5 Department of Metabolism and Nutrition, Institute of Food Science, Technology and Nutrition (ICTAN-CSIC), José Antonio Nováis 10, 28040 Madrid, Spain; jara.perez@ictan.csic.es; 6 CIBER of Diabetes and Associated Metabolic Diseases (CIBERDEM), Carlos III Health Institute (ISCIII), 28029 Madrid, Spain; 7 Microbiota and Vascular Biology Laboratory, IdISSC, c/Prof. Martín Lagos Street, 28040 Madrid, Spain; mgomezgarre@salud.madrid.org; 8 Biomedical Research Networking Center in Cardiovascular Diseases (CIBERCV), Monforte de Lemos Avenue 3–5, 28029 Madrid, Spain; 9 Department of Physiology, Faculty of Medicine, Complutense University of Madrid, Plaza Ramón y Cajal s/n, 28040 Madrid, Spain

**Keywords:** grape antioxidant dietary fiber, non-extractable polyphenol–fiber matrix, colonic barrier integrity, proliferation/cell-cycle, apoptosis, Hsp27, AMPK-mTOR-ACC1–CPT1 signaling

## Abstract

The colonic epithelium renews rapidly and must balance proliferation with apoptosis to preserve barrier integrity. We investigated whether grape antioxidant dietary fiber (GADF), a grape pomace-derived dietary fiber matrix naturally rich in high molecular weight non-extractable polyphenols, modulates barrier integrity, through proliferation/cell cycle and apoptosis. To gain mechanistic insight, we examined the role of heat-shock proteins (Hsps), and AMP-activated protein kinase (AMPK)–mTOR–lipid-metabolism signaling in healthy proximal colon. Male Wistar rats received either a cellulose-based control diet or an isoenergetic diet where cellulose was replaced with 5% GADF for four weeks. Morphometric analysis, immunohistochemistry, Western blotting, TUNEL, and caspase activity assays quantified cell cycle, apoptotic, Hsps, and metabolic pathways. GADF strengthened the epithelial barrier, increasing goblet cells, occludin, and ZO-1, while reducing crypt depth. Proliferation was suppressed, as indicated by reduced PCNA, cyclins E and D1, and higher p-p53^Ser392^, p21^Cip1/Waf1^, and p27^Kip1^ levels, consistent with G1 arrest. Apoptosis was attenuated, with increased mitochondrial Bcl-2/Bax and Bcl-xL/Bax ratios, lower cytosolic cytochrome c and apoptosis-inducing factor (AIF), and reduced caspase-9 and caspase-3 activities. Hsp27, but not Hsp70, was selectively induced. GADF activated AMPK and p-Raptor, enhanced ACC1 phosphorylation and CPT1, and supported a shift toward fatty acid β-oxidation. Correlation analysis revealed a strong association between Hsp27 and p-p53^Ser392^, suggesting potential links between barrier proteins and metabolic pathways. In conclusion, GADF preserves barrier integrity and redirects metabolism via AMPK–Hsp27 signaling, thereby promoting colonic homeostasis. These findings highlight grape pomace as a sustainable source of functional ingredients for nutritional strategies to reinforce epithelial defenses and reduce disease risk.

## 1. Introduction

Colonic epithelium undergoes rapid renewal, with crypt-base stem cells driving proliferation, differentiation, and migration, while apoptotic shedding at the lumen maintains barrier integrity [1,2]. This dynamic turnover is essential for sustaining the architecture of intestinal barrier tight junctions and their selective permeability [3]. An imbalance between proliferation and apoptosis can compromise epithelial differentiation, weaken barrier function, and predispose to inflammation and neoplasia [4]. Cell cycle checkpoints, particularly the G_1_/S transition, are closely integrated with metabolic sensing pathways that adjust proliferation rates according to nutrient and energy availability, linking epithelial renewal to metabolic signals [5]. Among these, heat-shock proteins (Hsps) are critical modulators of epithelial homeostasis that intersect with both cell-cycle control and metabolic signaling [6,7]. In the colon, Hsp27 and Hsp70 act as stress-inducible chaperones that preserve proteostasis, stabilize the actin cytoskeleton, and support tight-junction organization, thereby limiting paracellular permeability [8,9]. Beyond barrier maintenance, Hsp27/Hsp70 are involved in G_1_/S regulation [10]. Hsp27 modulates p53 activity and thereby regulates the cyclin-dependent kinase (CDK) inhibitors p21^Cip1/Waf1^ and p27^Kip1^, influencing cell cycle progression and cell survival [11]. These coordinated chaperone effects couple stress adaptation to controlled epithelial proliferation.

By contrast, AMP-activated protein kinase (AMPK) is the canonical cellular energy sensor that coordinates lipid and carbohydrate metabolism, cellular proliferation, and stress responses. In epithelial cells, AMPK phosphorylates acetyl-CoA carboxylase-1 (ACC1), reducing malonyl-CoA levels and relieving inhibition of carnitine palmitoyltransferase-1 (CPT1), the rate-limiting enzyme in mitochondrial β-oxidation [12]. This AMPK–ACC1–CPT1-driven metabolic shift toward fatty acid oxidation supports ATP production, sustains tight-junction assembly, and maintains the physiological hypoxic microenvironment of the lumen that limits barrier permeability and pathogen expansion [13,14,15]. Disruption of this axis has been linked to increased intestinal permeability, dysbiosis, and inflammatory susceptibility, whereas activation by dietary polyphenols or short-chain fatty acids (SCFAs) enhances epithelial integrity [16,17,18]. Bidirectional crosstalk links Hsps with AMPK signaling. Energy stress can activate AMPK, which signals through p38 mitogen-activated protein kinase/MAPK-activated protein kinase 2 (p38–MK2) to phosphorylate Hsp27, enhancing its chaperone and actin-capping activities that fortify junctional integrity [19]. Conversely, Hsp27 helps sustain mitochondrial function and redox balance, facilitating AMPK-driven reliance on β-oxidation and reducing ROS burden [7,20]. Together, these findings highlight a diet–chaperone–metabolism axis relevant to colonic health [21,22,23,24,25,26].

Diets rich in plant-derived bioactives are increasingly appreciated as modulators of intestinal homeostasis and long-term colonic health. Nutritional strategies, including polyphenols, prebiotic fibers, and specific commensals, have been reported to independently induce Hsp27 or activate AMPK, thereby strengthening barrier function. Among these, grape antioxidant dietary fiber (GADF), a winemaking by-product combining insoluble fiber bound to polyphenols such as catechins, and flavonols and, particularly, high molecular weight proanthocyanidins (belonging to the so-called non-extractable polyphenols), has emerged as a multifunctional dietary component capable of influencing epithelial proliferation, oxidative stress, and metabolic signaling in the colon [27,28]. Most polyphenols present in GADF, particularly the non-extractable ones, resist upper gastrointestinal digestion, reaching the colon intact, where microbial fermentation releases phenolic metabolites that have been shown to be bioavailable and with biological activities. This process promotes the growth of beneficial bacteria (*Lactobacillus* and *Bifidobacterium* spp.), enhances SCFAs production, and facilitates polyphenol utilization [29,30]. In healthy rats, GADF shifted the glutathione redox balance toward a more reduced state [31], inhibited mitochondrial apoptosis by upregulating Bcl-2 and Bcl-xL [32], and induced epithelial hypoplasia in the distal colon [33]. In cancer models, GADF suppressed tumorigenesis by inducing G_1_ cell-cycle arrest via downregulation of cyclin D [34] and/or through inhibition of Akt/mTOR signaling [35]. Grape seed extract, a related matrix, upregulated p21^Cip1/Waf1^, leading to similar G_1_ arrest [36]. GADF also induced apoptosis by stabilization of tumor suppressor proteins such as p53 [37], and upregulation of pro-apoptotic Bcl-2 family members [38,39].

Although these findings support the potential of GADF to modulate epithelial turnover, redox homeostasis, microbial ecology, and key intracellular signaling pathways in distal colon and cancer models, where its activity has been mainly linked to antioxidant defense and apoptosis regulation under pathological conditions, its effects in the healthy proximal colon, where epithelial renewal is most active, remain largely unknown. In contrast, our present work focuses on the healthy proximal colon, revealing novel associations between GADF and physiological mechanisms that sustain epithelial integrity. Therefore, the objective of this study was to evaluate the effects of GADF on colonocyte turnover, proliferation/cell cycle progression, apoptosis, cytoprotection via Hsps, and the AMPK–mTOR–lipid metabolism axis in the proximal colon of healthy rats. Understanding how GADF sustains colonic homeostasis will help us to valorize grape pomace as a sustainable functional ingredient and also to identify nutritional strategies that reinforce epithelial barrier integrity, preventing inflammation and disease.

## 2. Results

### 2.1. Effects of GADF on Proximal Colon Morphology and Barrier Integrity Markers

To investigate the impact of GADF on proximal colonic structure and barrier integrity, we first analyzed morphological and histological parameters. The main effects of GADF are summarized in Table 1, with representative histological and immunohistochemical images shown in Supplementary Table S1. GADF consumption enhanced barrier features, increased colon length (+17.8%), PAS-positive goblet cells (+21.8%), and the levels of tight-junction proteins occludin (+52.3%) and ZO-1 (+59.6%). Additionally, structural remodeling was observed, with a reduction in mucosal thickness (−6.5%) and crypt depth (−15.4%), whereas colon weight and crypt density remained unchanged.

### 2.2. GADF Modulates Proliferation and Cell Cycle Regulators in Proximal Colon Epithelium

**Figure 1:**
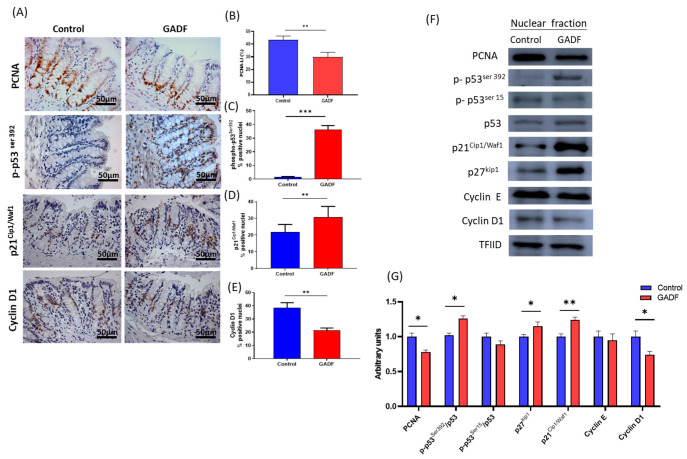
Effects of GADF on cell cycle regulators in the proximal colonic epithelium. (**A**) Representative immunohistochemical staining of PCNA, p-p53^Ser392^, p21^Cip1/Waf1^, and cyclin D1 in control and GADF groups (positive nuclei stained brown); scale bar = 50 µm. Images taken at 200× magnification. Representative fields are shown (**B**–**E**) Quantification of positive nuclei (%) (*n* = 10). (**F**) Western blot analysis of nuclear fractions for PCNA, p-p53^Ser392^, p-p53^Ser15^, p27^Kip1^, p21^Cip1/Waf1^, and cyclins E and D1 proteins (TFIID as loading control) (unpaired two-tailed Student’s *t*-test). (**G**) Densitometric quantification of nuclear proteins expressed as arbitrary units, calculated as the ratio of target band intensity to its corresponding loading control, normalized to the control mean (*n* = 3) (Mann–Whitney U test). Data are presented as mean ± SD. Statistical significance vs. control: * *p* < 0.05; ** *p* < 0.01; *** *p* < 0.001).

Given the importance of controlled proliferation in maintaining epithelial homeostasis, we next investigated the impact of GADF on key cell-cycle regulators (Figure 1).

Immunohistochemical staining confirmed these findings, showing a marked reduction in nuclear PCNA positivity in epithelial cells of GADF rats (−30.7%, *p* < 0.0001), accompanied by enhanced signals for p-p53^Ser392^ (+973.1%, *p* < 0.0001) and p21^Cip1/Waf1,^ (+40.3%, *p* < 0.01), and lower cyclin D1 levels (−44.4%, *p* < 0.01). Consistently, Western blot analysis demonstrated a significant downregulation of PCNA, cyclin D1, and E (−15% to −26%, *p* < 0.05), paralleled by increased levels of p-p53^Ser392^, p27^Kip1,^ and p21^Cip1/Waf1^ (−12% to −26%, *p* < 0.01), while no changes were observed in p-p53^Ser15^ levels. Together, these changes indicated a G1 phase arrest in epithelial cells.

### 2.3. GADF Attenuates Apoptosis in Proximal Colonic Mucosa

**Figure 2:**
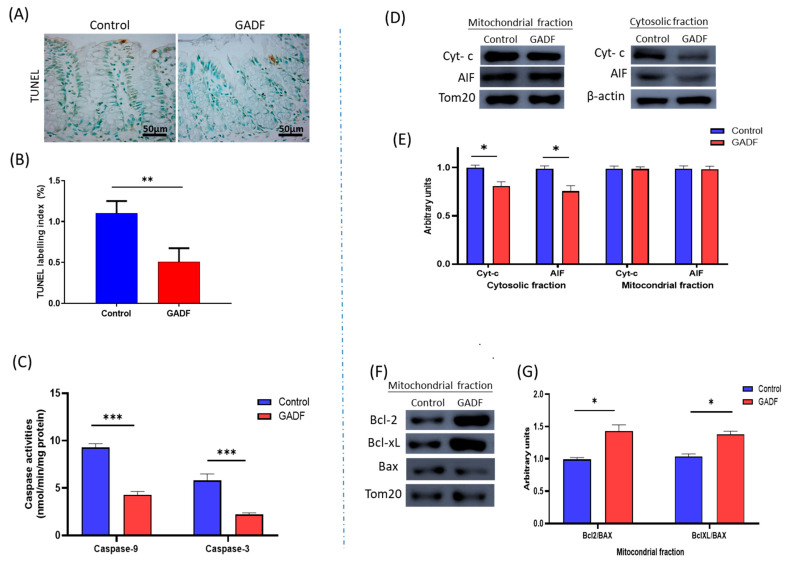
GADF attenuates apoptosis in the proximal colonic mucosa. (**A**) Representative TUNEL-stained sections (positive nuclei stained brown); Scale bar = 50 µm. Images taken at 200× magnification. Representative fields are shown (**B**) Quantification of TUNEL-LI (*n* = 10). (**C**) Caspase-9 and caspase-3 activities (*n* = 10) (unpaired two-tailed Student’s *t*-test). (**D**) Western blot analysis of cytosolic and mitochondrial fractions showing cytochrome and AIF (β-actin and TOM20 as loading controls). (**E**) Densitometric quantification of nuclear proteins expressed as arbitrary units, calculated as the ratio of target band intensity to its corresponding loading control, normalized to the control mean (*n* = 4) (Mann–Whitney U test). (**F**) Western blot of mitochondrial Bcl-2, Bcl-xL, and Bax. (**G**) Ratios of Bcl-2/Bax and Bcl-xL/Bax (*n* = 4). Data are mean ± SD. Statistical significance vs. control: * *p* < 0.01; ** *p* < 0.001; *** *p* < 0.0001.

To investigate whether GADF influenced epithelial survival, we next assessed key regulators of apoptosis in the proximal colonic mucosa (Figure 2). GADF intake markedly attenuated apoptosis, as reflected by a 54.4% reduction in TUNEL-LI (*p* < 0.01). Consistently, caspase-9 (−53.6%, *p* < 0.01) and caspase-3 (−62.2%, *p* < 0.01) activities were significantly lower in GADF rats compared with controls. At the cytosolic level, the release of cytochrome c (−21.0%, *p* < 0.05) and AIF (−19.0%, *p* < 0.05) was reduced, without significant changes in their mitochondrial content. In parallel, mitochondrial levels of the anti-apoptotic proteins Bcl-2 and Bcl-xL were increased, whereas Bax levels remained unchanged, leading to higher Bcl-2/Bax and Bcl-xL/Bax ratios (+42.9% and +37.9%, respectively; *p* < 0.05). Together, these findings indicated that GADF reduced mitochondrial outer membrane permeabilization and thereby suppressed epithelial apoptosis.

### 2.4. GADF Induces the Cytoprotective Chaperone Hsp27 in Proximal Colonic Mucosa

**Figure 3:**
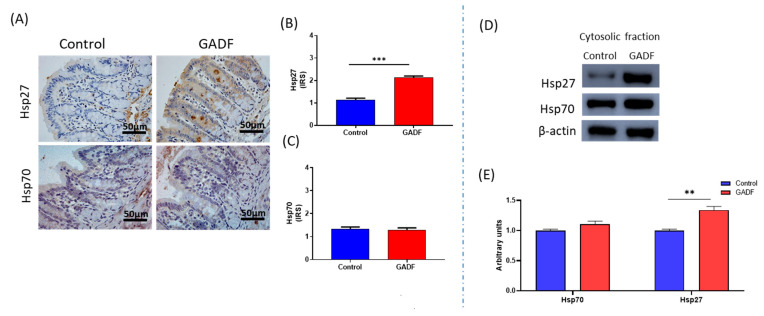
Induction of Hsp27 by GADF. (**A**) Representative immunohistochemical staining of Hsp27 and Hsp70 in colonic sections from control and GADF-fed rats (cytoplasmic brown color); scale bar = 50 µm. Images taken at 200× magnification. Representative fields are shown (**B**,**C**) Quantification of IRS for Hsp27 and Hsp70 (*n* = 10). (**D**) Western blot analysis of cytosolic fractions for Hsp27 and Hsp70, with β-actin as loading control. (**E**) Densitometric quantification of nuclear proteins expressed as arbitrary units, calculated as the ratio of target band intensity to its corresponding loading control (β-actin), normalized to the control mean (*n* = 4) (Mann–Whitney U test). Data are presented as mean ± SD. Statistical significance vs. control: ** *p* < 0.001; *** *p* < 0.0001.

In parallel with its anti-apoptotic effects, GADF modulated cytoprotective Hsps in the proximal colon (Figure 3). Supplementation increased Hsp27, as shown by stronger immunohistochemical staining and higher cytosolic protein levels in Western blotting (+83.3%, *p* < 0.001, +34.4%, *p* < 0.01), whereas Hsp70 expression remained unchanged. These findings indicated a selective induction of Hsp27 by GADF.

### 2.5. GADF Activates AMPK and Shifts Lipid Metabolism Toward β-Oxidation

**Figure 4:**
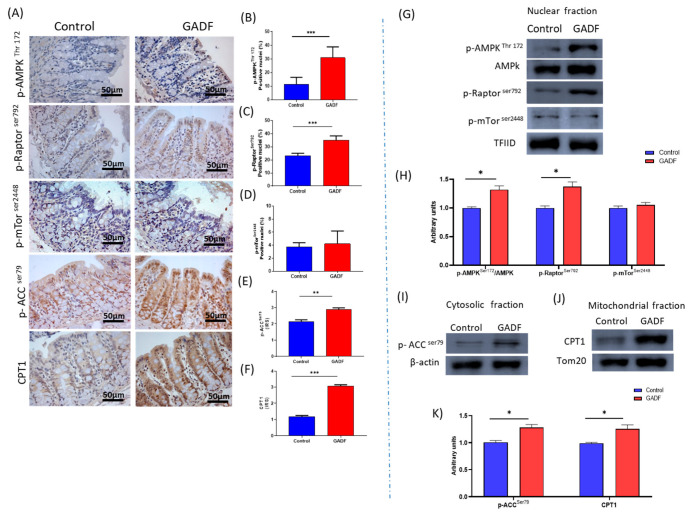
GADF activates AMPK via Raptor, promoting a metabolic shift toward fatty acid β-oxidation. (**A**) Representative immunohistochemical staining of p-AMPK^Thr172^, p-Raptor^Ser792^, mTOR^Ser2448^, p-ACC ^Ser79^, and CPT1 in control and GADF groups (brown positive staining); scale bar = 50 µm. Images taken at 200× magnification. Representative fields are shown (**B**–**D**). Quantification of immunopositive nuclei for p-AMPK^Thr172^, p-Raptor^Ser792^, and mTOR^Ser2448^. (**E**,**F**) IRS scores for p-ACC ^Ser79^ and CPT1 (*n* = 10). (**G**) Western blot analysis of nuclear fractions for p-AMPK^Thr172^, p-Raptor^Ser792^, and mTOR^Ser2448^, with TFIID as loading control (*n* = 3). (**H**) Densitometric quantification of nuclear proteins expressed as arbitrary units, calculated as the ratio of target band intensity to its corresponding loading control, normalized to the control mean (Mann–Whitney test). (**I**) Western blot of cytosolic fraction for p-ACC^Ser79^ with β-actin as loading control. (**J**) Western blot of mitochondrial fraction for CPT1 with TOM20 as loading control (*n* = 3). (**K**) Densitometric quantification of p-ACC^Ser79^ and CPT1 proteins expressed as arbitrary units. Data are presented as mean ± SD. Statistical significance vs. control: * *p* < 0.01; ** *p* < 0.001; *** *p* < 0.0001.

Since AMPK signaling is a central regulator of energy balance and barrier integrity, we next investigated the impact of GADF on the AMPK–mTOR pathway and the downstream ACC1–CPT1 axis in the proximal colon (Figure 4). GADF activated AMPK signaling in the proximal colonic mucosa. Nuclear phosphorylation of p-AMPK^Thr172^ (+29.8% by IHC, +40% by WB) and p-Raptor^Ser792^ (+51.1% by IHC, +38.9% by WB), was markedly increased, while phosphorylation of mTOR^Ser2448^, remained unchanged. In parallel, downstream activation of p-ACC^Ser79^ (+34.5% by IHC, +23.3% by WB) and increased levels of CPT1 (+162% by IHC, +30% by WB) indicated a metabolic shift toward fatty acid β-oxidation. These findings indicated that GADF activated AMPK via Raptor in nuclear compartment and promoted a metabolic shift from lipid synthesis toward fatty acid β-oxidation through the ACC1–CPT1 axis.

### 2.6. Correlation Heatmap of Epithelial and Molecular Markers

**Figure 5:**
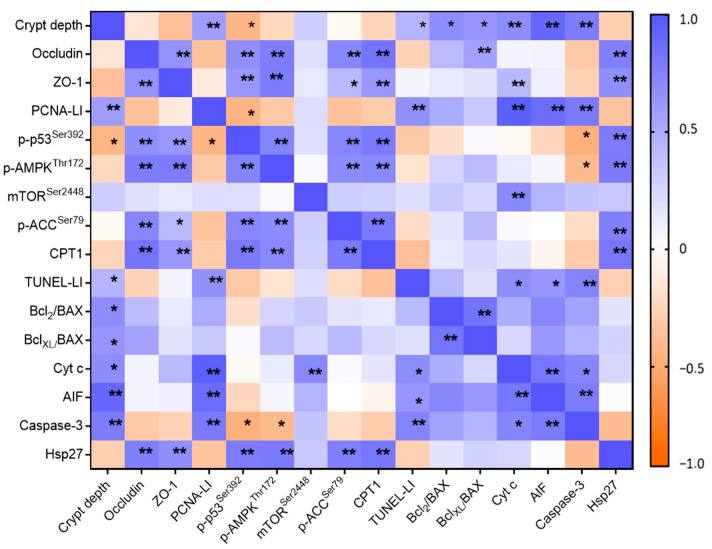
Correlation between epithelial morphology, barrier proteins, proliferative/cell-cycle markers, apoptotic markers, Hsp27, and metabolic signaling. Heatmap of Spearman’s correlation coefficients. The color scale indicates direction and strength (blue, positive; orange, negative; −1 to +1). Asterisks denote statistical significance (* *p* < 0.05; ** *p* < 0.01).

To explore potential functional relationships among the mechanisms studied, we performed Spearman’s correlation analysis (Figure 5).

GADF-associated changes revealed that crypt depth correlated positively with proliferative and apoptotic markers, and inversely with p-p53^Ser392^. Tight junction proteins (occludin, ZO-1) were strongly linked to p-p53^Ser392^, AMPK signaling, and Hsp27. The proliferation marker PCNA-LI showed a positive correlation with apoptotic mediators and a negative association with p-p53^Ser392^, whereas apoptotic proteins were strongly interrelated. Notably, Hsp27 displayed extensive associations with barrier proteins, stress signaling, and metabolic regulators, suggesting a potential coordinating role in cytoprotection and barrier maintenance.

## 3. Discussion

The present study shows that, in healthy rats, dietary GADF modulates proximal colonic homeostasis through multiple coordinated mechanisms. The main outcomes were as follows: (1) reinforcement of barrier integrity, evidenced by increased goblet-cell numbers and higher occludin and ZO-1 levels; (2) slower epithelial turnover with reduced crypt depth, decreased proliferation, G1-phase cell-cycle arrest, and lower apoptosis; (3) selective upregulation of Hsp27, but not Hsp70, supporting cytoprotection and colonic epithelial integrity; and (4) a shift in lipid metabolism toward β-oxidation via AMPK and the ACC1–CPT1 pathway. Together, these responses promoted an epithelial phenotype characterized by improved barrier integrity and metabolic efficiency, highlighting the potential of GADF as a functional food ingredient for sustaining intestinal health.

GADF is a patented ingredient obtained from grape pomace [40], a major by-product of wine production. Its valorization not only adds sustainability by reducing agricultural waste but also provides a source of dietary fiber and non-extractable polyphenols, giving rise to an antioxidant dietary-fiber matrix that explains its integrative effects [41,42,43]. In this study, cellulose served as an inert reference fiber and was completely replaced entirely by GADF, allowing the observed effects to be attributed to its complex matrix of dietary fiber and polyphenols, most of them of polymeric nature. It has been shown that matrices rich in both the so-called non-extractable polyphenols (associated by several linkages with food matrix or of high molecular weight) and dietary fiber have a specific metabolic fate. In particular, they exhibit a delayed release of microbial-derived bioactive phenolic metabolites, ensuring their circulation during prolonged times, as well as a mutual enhancement of the transformation of both dietary fiber and polyphenols by colonic microbiota [44]. Indeed, recent evidence indicates that non-extractable polyphenol–fiber conjugates exhibit more sustained bioactivity than their free counterparts and are promising functional food ingredients [45,46]. And consistent with this, depletion of bound polyphenols reduces SCFA production and antioxidant potential [47], whereas their association with insoluble fiber enhances microbial fermentation and strengthens antioxidant defenses [48].

The present results demonstrate that GADF improved colonic barrier function by increasing colon length, goblet cell numbers, and occludin and ZO-1 levels, while reducing mucosal thickness and crypt depth. The parallel increase in tight junction proteins and goblet cells suggested that GADF strengthens barrier integrity at both the structural and secretory levels. These findings are consistent with earlier observations [33] and indicate reinforcement of the epithelial barrier. Furthermore, our previous work showed that GADF promotes *Lactobacillus* growth [29,49], consistent with evidence that species such as *L. gasseri*, *L. plantarum*, and *L. rhamnosus* enhance epithelial integrity by upregulating occludin and ZO-1 and stimulating mucin (MUC2) secretion [17,50]. Barrier reinforcement likely results from microbial fermentation of fiber–polyphenol complexes, which release bioactive metabolites and enhance SCFAs production, thereby strengthening epithelial integrity [51,52,53]. The previously reported improvement in colonic redox status [31,32] supports our current findings, linking antioxidant protection with barrier preservation. Converging evidence indicates that GADF’s redox activity is key to barrier protection, integrating antioxidant and microbiota-driven mechanisms to support colonic balance.

A key finding of this study is the coordinated reduction in colonocyte proliferation and apoptosis, indicating slower epithelial turnover. GADF lowered PCNA-LI, cyclin D1, and cyclin E levels while increasing p-p53^Ser392^, p21^Cip1/Waf1^**,** and p27^Kip1^, consistent with G1 arrest. Specifically, GADF promoted selective phosphorylation of p53 at Ser392, but not Ser15. Crypt depth correlated positively with PCNA-LI but inversely with p-p53^Ser392^. These results indicate that p53-driven cell-cycle control and epithelial maintenance predominate over canonical DNA-damage signaling [54,55]. Modulation of the G1/S checkpoint by dietary polyphenols is a recognized mechanism for preserving epithelial integrity and preventing uncontrolled proliferation [56]. In this context, p27^Kip1^ cooperates with p21^Cip1/Waf1^ to inhibit CDK2/CDK4 activity, reinforcing the reduction in cyclin-D/E–dependent S-phase entry and aligning with the observed decrease in crypt depth. Similar effects have been reported for polyphenols from edible seaweed and *Hippophae rhamnoides*, green tea, and cocoa, all inducing G_0_/G_1_ arrest and activating p53/p21 signaling in colonocytes [57,58,59,60,61].

In parallel, GADF attenuated epithelial cell death through both caspase-dependent and AIF-mediated caspase-independent routes. TUNEL-LI, caspase-3 and caspase-9 activities, and cytosolic cytochrome c and AIF decreased, while mitochondrial Bcl-2 and Bcl-xL increased, leading to higher Bcl-2/Bax and Bcl-xL/Bax ratios, findings consistent with limited mitochondrial outer-membrane permeabilization (MOMP). The shift toward an antiapoptotic profile reduces Bax-dependent pore formation and the cytosolic efflux of cytochrome c and AIF, which in turn stabilizes the mitochondrial outer membrane and maintains a low-turnover epithelial state [62]. Similar mitochondria-protective patterns have been reported in the distal colon under GADF treatment [32]. Positive correlations linking the TUNEL-LI, crypt depth, and PCNA-LI, indicate that GADF induced a coordinated lowering of epithelial turnover that helps maintain the mucosa. Consistently, p-p53^Ser392^ correlated negatively with PCNA-LI and caspase-3 activity, underscoring its central role in restraining proliferation and apoptosis while supporting epithelial maintenance. In contrast, in the distal colon, GADF suppresses apoptosis without affecting proliferation, acting through mitochondrial stabilization and antioxidant reinforcement [32,63]. These effects reflect segment-specific demands: the proximal colon conserves resources by slowing proliferation and reinforcing barrier integrity, while the distal colon maintains renewal under higher microbial and oxidative stress [48,64]. Thus, GADF modulates epithelial turnover in a region-specific manner, strengthening barrier function in the proximal colon.

A distinctive result of this study was the selective induction of Hsp27, with no significant change in Hsp70. Hsp27 correlated positively with tight-junction proteins and p-p53^Ser392^, but not with apoptotic markers, supporting its role in cytoskeletal stabilization and barrier reinforcement rather than direct apoptosis inhibition. Consistently, Hsp27 stabilizes actin, supports tight junctions, and regulates stress responses to preserve barrier homeostasis [65]. Importantly, recent nutritional studies demonstrate that dietary supplementation can selectively activate Hsp27 to promote intestinal protection. Non-digestible fibers such as xylobiose and partially hydrolyzed guar gum upregulate Hsp27 in intestinal epithelial cells, enhancing tight junction integrity and cytoskeletal stabilization [25,26], while the probiotic *Akkermansia muciniphila* similarly enhances barrier function via Hsp27 phosphorylation [66]. The activation of Hsp27 by GADF emerges as a promising nutritional target linking dietary signals with cytoskeletal stabilization, tight-junction reinforcement, and epithelial repair.

Our findings indicate that metabolic reprogramming is a key mechanism through which GADF stabilizes epithelial homeostasis. AMPK is a master regulator of energy status and proliferation [12,13], and its deficiency accelerates colorectal tumorigenesis [67]. GADF markedly increased p-AMPK^Thr172^ and p-Raptor^Ser792^, while leaving mTOR^Ser2448^ phosphorylation unchanged, suggesting selective inhibition of mTORC1 through Raptor rather than direct mTOR suppression [68,69]. These observations align with evidence that AMPK activation silences mTORC1 via Raptor/TSC2 without necessarily reducing mTOR phosphorylation, as shown in intestinal differentiation [70].

In addition, GADF reinforced AMPK signaling through its downstream effectors ACC1 and CPT1, thereby promoting a metabolic shift toward fatty acid β-oxidation [71]. Positive correlations between p-AMPK^Thr172^, p-ACC^Ser79^, and CPT1 confirmed a coordinated AMPK–ACC1–CPT1 response. This metabolic shift confers dual benefits by enhancing ATP efficiency and reducing ROS generation, thereby alleviating oxidative stress. Given that colonocytes naturally depend on fatty acid and butyrate oxidation, this reprogramming likely improved energetic balance and reduced the requirement for rapid turnover, directly linking metabolism to epithelial stability [72,73]. Comparable effects have been documented in other polyphenol-rich matrices. Grape pomace extracts mitigate metabolic dysregulation in diet-induced models and have been reported to modulate energy-metabolism pathways [68,74,75], while quercetin increases CPT1 and reduces ACC1 through AMPK activation, promoting β-oxidation in vivo [17]. Collectively, these parallels reinforce the idea that GADF engages the AMPK–Raptor–ACC1–CPT1 axis as part of a broader polyphenol-mediated metabolic regulation.

The modulation of the AMPK–p53 axis by GADF appears central to this integration. p-AMPK^Thr172^ correlated positively with p53^Ser392^, placing p53 downstream of AMPK activation. Thus, AMPK can activate p53 and induce p21^Cip1/Waf1^, enforcing a G1 checkpoint [76], while p53 constrains glycolysis and promotes fatty-acid oxidation, thereby rewiring metabolism toward β-oxidation [77]. Consistently, p27^Kip1^ also increased, providing additional CDK inhibition that consolidates G1 control under energy stress [78]. In parallel, GADF revealed a positive correlation between Hsp27, p-AMPK^Thr172^, and ACC1/CPT1. This pattern favors ATP efficiency, preserves energy balance, and reduces ROS, while simultaneously engaging AMPK/p38–MK2/Hsp27 signaling, which stabilizes the cytoskeleton and reinforces tight junction integrity [19,79]. It also aligns data showing that resveratrol, quercetin, and curcumin strengthen barrier integrity and mitochondrial resilience via AMPK/Nrf2 and autophagy [80,81].

Collectively, our findings indicate that GADF fosters a low-proliferative, stress-resistant, and β-oxidation-oriented epithelial state. This phenotype is mediated by AMPK-driven metabolic reprogramming and Hsp27-dependent cytoprotection, which act together to reinforce barrier integrity. These coordinated mechanisms position GADF as a sustainable functional ingredient for intestinal health, exemplifying circular food-system strategies that transform winery by-products into bioactive resources.

## 4. Materials and Methods

### 4.1. GADF Preparation and Composition

GADF was obtained from red grapes (*Vitis vinifera* var. *Cencibel*, La Mancha region, Spain) as previously described [40]. Its main constituents are dietary fiber (73.5 ± 0.8%; insoluble 58.0 ± 0.8%, soluble 15.5 ± 0.1%, both determined by the indigestible fraction method), total polyphenols (19.7 ± 0.2%, including 15% of non-extractable polyphenols, in particular, polymeric proanthocyanidins). Proximate analysis showed the presence of protein (11.1 ± 0.5%), fat (7.7 ± 0.5%), and ash (5.3 ± 0.2%) [31,32]. In addition, LC/ESI-MS analyses have shown that GADF provides a complex mixture of bioactive phenolics, mainly phenolic acids, flavan-3-ols, flavonols, anthocyanins, and oligomeric to polymeric proanthocyanidins as the major fraction [43]. Consistent with this composition, its antioxidant capacity has been reported to reach 124.4 ± 0.3 μmol Trolox/g dry matter by the ABTS assay and 214.2 ± 38 μmol Trolox/g dry matter by the ORAC assay [31,32].

### 4.2. Animals and Experimental Design

Male Wistar rats (200–250 g; Harlan Ibérica, Barcelona, Spain) were housed at the Animal Experimentation Center, Faculty of Pharmacy, Complutense University of Madrid (registration No. ES-28079-0000085), under controlled conditions (22 ± 2 °C; 12 h light/dark cycle) with ad libitum access to food and water. All procedures were approved by Spanish regulatory authorities (PROEX124.3/25) and conducted in accordance with Directive 2010/63/EU and the ARRIVE guidelines. Environmental enrichment was provided throughout the feeding period, and animals were monitored daily; humane endpoints were applied according to institutional guidelines to minimize distress. After a 7-day acclimatization period, animals were randomly allocated into two groups (n = 10 per group): (1) Control, fed a standard diet containing cellulose as the sole dietary fiber source, and (2) GADF, fed an isoenergetic diet in which cellulose was replaced with 5% (*w*/*w*) GADF, corresponding to the recommended human dietary fiber intake [82]. Both diets were formulated and manufactured by Dyets Inc. (Bethlehem, PA, USA), and the feeding period lasted 4 weeks. At the end of the experiment, animals were anesthetized with ketamine (40 mg/kg) and xylazine (5 mg/kg) and euthanized by cervical dislocation. The proximal colon (first half of the colon) was excised, rinsed with ice-cold phosphate-buffered saline (PBS), and divided for analysis: tissue samples for histology and immunohistochemistry were fixed in 4% paraformaldehyde, embedded in paraffin, and sectioned at 4 μm, while mucosal scrapings for Western blot and biochemical assays were snap-frozen in liquid nitrogen and stored at −80 °C until use.

### 4.3. Subcellular Fractionation

The proximal mucosae were homogenized in a Potter-Elvehjem homogenizer with a Teflon/glass piston (ARPIVAL S.A., Madrid, Spain), using extraction buffer (210 mM mannitol, 70 mM sucrose, 5 mM HEPES, 1 mM EDTA pH 7.4) with protease inhibitor cocktail (Sigma Aldrich, Madrid, Spain). Homogenates were centrifuged at 800× *g* for 10 min. to pellet the nuclei and to obtain a supernatant with the cytosolic fraction. The supernatants were further centrifuged at 15,000× *g* for 15 min. at 4 °C to obtain the total cytosolic fraction. The cytosolic supernatants were filtered and then the mitochondria were pelleted by centrifugation at 14,000× *g* at 4 °C for 25 min. The mitochondrial pellets were resuspended in lysis buffer and centrifuged at 14,000× *g* at 4 °C for 25 min. The resulting supernatant was the mitochondrial fraction. The nuclei pellets were resuspended in nuclear extraction buffer (20 mM HEPES, 1.5 mM MgCl_2_, 0.42 M NaCl, 0.2 mM EDTA, 1 mM dithiothreitol, 25% glycerol pH = 7.4) containing protease inhibitors for 30 min. on ice with vortexing at 10 min. intervals at 4 °C. The nuclei homogenates were filtered through fine nylon mesh (pore size 75 μm) to remove any unbroken cells and connective tissue and centrifuged for 30 min. at 14,000× *g* at 4 °C to collect supernatant as nuclear fraction. Aliquot of all extracts and store at −80 °C until assay.

### 4.4. Western Blotting

Immunoblots were performed using colonic lysates and subcellular fractions. Protein extracts (DC Protein Assay Kit, Bio-Rad, Madrid Spain) from each sample were separated by SDS–PAGE and transferred to polyvinylidene fluoride membranes (GE Healthcare, Madrid, Spain). After blocking, membranes were incubated overnight at 4 °C with primary antibodies against p21^WAF1/Cip1^ (F-5, sc-6246), p27^Kip1^ (F-8, sc-1641), cyclin E (HE12, sc-247), cyclin D1 (A-12, sc-8396), Bax (2D2, sc-20067), Bcl-2 (C-2, sc-7382), Bcl-xL (H-5, sc-8392), cytochrome c (A-8, sc-13156), AIF (E-1, sc-13116), Hsp70 (W27, sc-24), Hsp27 (F-4, sc-13132), AMPKα1/2 (D-6, sc-74461), p-AMPK^Thr172^ (sc-33524), CPT1 (E-7, sc-393070), PCNA (PC10, sc-56), β-actin (C4, sc-47778; cytosolic control), TOM20 (F-10, sc-17764; mitochondrial control), and TFIID (58C9, sc-421; nuclear control) from Santa Cruz Biotechnology (anti-mouse; Quimigen, Madrid, Spain); p53 (DO-7, MA5-12557), p-p53^Ser392^ (44–640 G), and p-mTOR^Ser2448^ (44–1125 G) from Invitrogen (Thermo Fisher Scientific, Madrid, Spain; anti-rabbit); and p-Raptor^Ser792^ (#2083) and p-ACC^Ser79^ (#3661) from Cell Signaling Technology (anti-rabbit; Danvers, MA, USA). Then, membranes were incubated with peroxide-conjugated secondary antibodies (anti-mouse or anti-rabbit, 1:5000 dilution) for 1 h at room temperature. Band detection was performed using enhanced chemiluminescence (ECL Select; GE Healthcare, Madrid, Spain) according to the manufacturer’s instructions, and exposure times were standardized across blots to avoid signal saturation. Densitometric analysis was conducted using ImageQuant 5.0 (GE Healthcare, Madrid, Spain) under identical threshold settings and batch processing for all membranes. Protein band intensities were quantified by investigators blinded to the experimental groups and expressed as arbitrary units relative to the corresponding loading control.

### 4.5. Histological Procedure Staining

Proximal colonic samples were fixed in 10% formaldehyde, embedded in paraffin, and cut into 3 µm-thick sections. The sections were stained with hematoxylin–eosin (H&E) and periodic acid Schiff (PAS) for histological analysis. Images were captured with a Leica DM LB2 light microscope and a Leica DFC 320 camera (Leica Microsystems S.L.U., Madrid, Spain) and analyzed with ImageJ software Fiji (ImageJ, version 1.54j; National Institutes of Health, Bethesda, MD, USA). Crypt depth was assessed on H&E-stained sections using ImageJ software. Measurements were obtained by tracing a straight line from the luminal opening to the crypt base (µm), focusing only on crypts with an open longitudinal axis. The level of neutral mucin glycoprotein in the tissue was assessed by PAS staining and calculated as the number of PAS-positive cells per crypt.

### 4.6. Immunohistochemistry

Paraffin-embedded sections were deparaffinized, rehydrated in a graded ethanol series. After citrate antigen retrieval and quenching of endogenous peroxidase, sections were incubated with anti-p21^WAF1/Cip1^ (F-5, sc-6246), anti-cyclin D1 (A-12, sc-8396), anti-Hsp70 (W27, sc-24), anti-Hsp27 (F-4, sc-13132), anti-p-AMPK^Thr172^ (sc-33524), anti-PCNA (PC10, sc-56) (Santa Cruz Biotechnology. Quimigen. Madrid. Spain); anti-p-p53^Ser392^ (44–640 G), and anti-p-mTOR^Ser2448^ (44–1125 G) (Invitrogen, Thermo Fisher Scientific, Madrid, Spain), and anti p-Raptor^Ser792^ (#2083) (Cell Signaling Technology, Danvers, MA, USA) primary antibodies overnight at 4 °C. The color reaction was developed with polymerized horseradish peroxidase-conjugated with secondary antibody and counterstaining with Hematoxylin. Positive and negative controls were included in each batch to confirm staining specificity. Image acquisition and analysis were standardized across samples: ten non-overlapping fields per section were captured at 400× magnification using identical exposure and threshold settings Fiji (ImageJ, version 1.54j; National Institutes of Health, Bethesda, MD, USA). Regions of interest (ROIs) were manually defined to include well-oriented crypts, and batch processing was applied to ensure consistent quantification across animals. For quantification of the positive nuclei number and PCNA labelling index (LI, %), at least twenty perpendicular well-oriented crypts were examined in each animal under light microscopy at 200 x magnification. The LI was calculated as the ‘number of positive nuclei × 100/total number of cells/crypt column height’. Immunoreactivity was quantified in 10 fields per section per rat using the immunoreactivity score (IRS), categorized as weak (1), moderate (2), diffuse (3), or intense (4). IRS was evaluated by two independent, blinded observers (>90% concordance).

### 4.7. Apoptosis Detection (TUNEL Assay)

Apoptosis was detected using the terminal deoxynucleotidyl transferase dUTP nick end labeling (TUNEL) procedure [83]. Briefly, the sections of proximal colon were deparaffinized, rehydrated, and permeabilized with proteinase K. After quenching, sections were incubated with terminal deoxynucleotidyl transferase reaction (TdT) mixture. The incorporated biotinylated nucleotides were detected by streptavidin-HRP. Negative controls consisted of samples of colon mucosa incubated without the TdT enzyme and biotin dUTP. Thereafter, sections were incubated with diaminobenzidine (DAB) until color development, mounted after dehydration, and counterstained with methyl green. The TUNEL labeling index (LI, %) was calculated as (number of apoptotic cells × 100)/total number of cells per crypt column height. For quantification, at least 50 well-oriented perpendicular crypts were examined and counted per animal at 400× magnification using a Leica DM LB2 microscope equipped with a digital Leica DFC 320 camera (Leica Microsystems S.L.U., Madrid, Spain). Apoptotic cells were quantified by a blinded investigator.

### 4.8. Caspase Activity Assays

Caspase-9 and caspase-3 activities were determined using colorimetric assay kits (Biovision Research Products, Madrid, Spain) with substrates Ac-LEHD-pNA and Ac-DEVD-pNA, respectively. Absorbance was measured at 405 nm, and activities were expressed as nmol pNA/min/mg protein (SpectroStar Nano, BMG, LABTECH, Offenburg, Germany).

### 4.9. Statistical Analysis

Data are presented as mean ± SD. Normality was assessed with the Shapiro–Wilk test and homoscedasticity with Levene’s test. For two-group comparisons, unpaired two-tailed Student’s *t*-tests were applied to normally distributed data, whereas the Mann–Whitney U test was used for non-normally distributed data. Effect sizes with 95% confidence intervals were calculated for all comparisons. Associations between continuous variables were analyzed using Spearman’s rank correlation, as appropriate, and coefficients are reported as ρ with two-sided *p* values. A *p* value < 0.05 was considered statistically significant. Statistical analyses were performed using SPSS v28 (IBM, Chicago, IL, USA), and graphs were generated with GraphPad Prism v8 (GraphPad Software, San Diego, CA, USA). An a priori power analysis (G*Power v3.1) based on expected 25–30% differences in histological and apoptotic parameters (SD ≈ 15%, α = 0.05, power = 0.8) justified a sample size of n = 8 animals per group; n = 10 were used to account for possible losses.

## 5. Conclusions

GADF profoundly reshaped proximal colon homeostasis by combining the structural properties of dietary fiber with the bioactivity of non-extractable polyphenols. In healthy rats, GADF reinforced epithelial barrier integrity, reduced proliferation and apoptosis to slow epithelial turnover, redirected energy metabolism toward β-oxidation via AMPK activation, and selectively induced Hsp27 to strengthen cytoprotection. Altogether, these coordinated effects established a stable, stress-resistant epithelial phenotype and highlight GADF as a sustainable functional ingredient with the potential to improve intestinal health.

## Figures and Tables

**Table 1:** Effects of GADF on colonic morphology and barrier markers in rats.

	Control	GADF	*p*
Colon weight (g)	2.63 ± 0.63	2.67 ± 0.64	NS
Length (cm)	13.67 ± 2.01	16.14 ± 1.68	<0.01
Mucosal thickness (µm)	255.00 ± 19.44	238.20 ± 30.93	<0.01
Crypt depth (µm)	225.91 ± 19.42	190.90 ± 28.95	<0.001
Crypt density (crypts/mm)	17.00 ± 2.94	16.7 ± 2.41	NS
PAS (positive cells/crypt)	21.50 ± 2.76	26.20 ± 2.90	<0.01
Occludin (IRS)	1.68 ± 0.33	2.56 ± 0.37	<0.01
ZO-1 (IRS)	0.99 ± 0.40	1.58 ± 0.49	<0.01

Values are expressed as mean ± SD (*n* = 10). Significantly different from control group (*p* < 0.05). NS: no significant difference between groups. (Student’s *t*-test or Mann–Whitney U test as appropriate).

## Data Availability

The dataset supporting this study is publicly available in the Zenodo repository under the Creative Commons Zero (CC0 1.0) license https://doi.org/10.5281/zenodo.17232503, accessed on 30 September 2025.

## Supplementary Materials

The following supporting information can be downloaded at: https://www.mdpi.com/article/10.3390/ijms262110564/s1.

## Abbreviations

ACC1	acetyl-CoA carboxylase 1
AIF	apoptosis-inducing factor
AMPK	AMP-activated protein kinase
Bax	Bcl-2 Associated X-protein
Bcl-2	B-cell lymphoma 2
Bcl-xL	B-cell lymphoma–extra-large
CPT1	carnitine palmitoyltransferase-1
CDK	cyclin-dependent kinase
Cyt c	cytochrome c
GADF	grape antioxidant dietary fiber
H&E	hematoxylin and eosin
Hsp27	heat shock protein 27
Hsp70	heat shock protein 70
Hsps	heat shock proteins
IHC	immunohistochemistry
IRS	immunoreactivity score
MAPK	mitogen-activated protein kinase
mTOR	mechanistic target of rapamycin
MOMP	mitochondrial outer-membrane permeabilization
ORAC	oxygen radical absorbance capacity
PAS	periodic acid–Schiff
PCNA	proliferating cell nuclear antigen
PCNA-LI	PCNA labeling index
p-ACC^Ser79^	ACC phosphorylated at serine 79
p-AMPK^Thr172^	AMPK phosphorylated at threonine 172
p-mTOR^Ser2448^	mTOR phosphorylated at serine 2448
p-p53^Ser392^	p53 phosphorylated at serine 392
p-Raptor^Ser792^	Raptor phosphorylated at serine 792
ROS	reactive oxygen species
SCFAs	short-chain fatty acids
TFIID	TFIID/TBP: TATA-binding protein
TOM20	translocase of outer mitochondrial membrane 20
TUNEL-LI	terminal deoxynucleotidyl transferase dUTP nick-end labeling index
WB	Western Blot
ZO-1	zonula occludens-1

## References

[1] Williams J.M., Duckworth C.A., Burkitt M.D., Watson A.J.M., Campbell B.J., Pritchard D.M. (2015). Epithelial Cell Shedding and Barrier Function: A Matter of Life and Death at the Small Intestinal Villus Tip. Vet. Pathol..

[2] Liu Y., Chen Y.G. (2020). Intestinal Epithelial Plasticity and Regeneration via Cell Dedifferentiation. Cell Regen..

[3] Díaz-Coránguez M., Liu X., Antonetti D.A. (2019). Tight Junctions in Cell Proliferation. Int. J. Mol. Sci..

[4] Choi J., Augenlicht L.H. (2024). Intestinal Stem Cells: Guardians of Homeostasis in Health and Aging amid Environmental Challenges. Exp. Mol. Med..

[5] Diehl F.F., Sapp K.M., Vander Heiden M.G. (2024). The Bidirectional Relationship between Metabolism and Cell Cycle Control. Trends Cell Biol..

[6] Binder M.J., Pedley A.M. (2023). The Roles of Molecular Chaperones in Regulating Cell Metabolism. FEBS Lett..

[7] Zou Y., Shi H., Liu N., Wang H., Song X., Liu B. (2023). Mechanistic Insights into Heat Shock Protein 27, a Potential Therapeutic Target for Cardiovascular Diseases. Front. Cardiovasc. Med..

[8] Zhang Y., Wang X., Wang S., Yan Z., Li C., Zheng Y., Cui L. (2020). Heat Shock Protein 27 Regulates the Inflammatory Response of Intestinal Epithelial Cells by the Nuclear Factor-ΚB Pathway. Dig. Dis. Sci..

[9] Cham C.M., Messer J.S., Lake J., Zhu X., Tao Y., He L., Weber C.R., Lin F., Dai Z., Tong J. (2022). Intestinal Epithelial Heat Shock Protein 25/27 Integrates Host and Microbial Drivers of Mucosal Restitution Following Inflammatory Injury. bioRxiv.

[10] Lechuga S., Marino-Melendez A., Naydenov N.G., Zafar A., Braga-Neto M.B., Ivanov A.I. (2024). Regulation of Epithelial and Endothelial Barriers by Molecular Chaperones. Cells.

[11] Lampros M., Vlachos N., Voulgaris S., Alexiou G.A. (2022). The Role of Hsp27 in Chemotherapy Resistance. Biomedicines.

[12] Hardie D.G. (2014). AMPK–Sensing Energy While Talking to Other Signaling Pathways. Cell Metab..

[13] Steinberg G.R., Carling D. (2019). AMP-Activated Protein Kinase: The Current Landscape for Drug Development. Nat. Rev. Drug Discov..

[14] Olivier S., Leclerc J., Grenier A., Viollet M.F.B., Tamburini J. (2019). AMPK Activation Promotes Tight Junction Assembly in Intestinal Epithelial Caco-2 Cells. Int. J. Mol. Sci..

[15] Zheng L., Kelly C.J., Colgan S.P. (2015). Physiologic Hypoxia and Oxygen Homeostasis in the Healthy Intestine. A Review in the Theme: Cellular Responses to Hypoxia. Am. J. Physiol. Cell Physiol..

[16] He J., Zhang P., Shen L., Niu L., Tan Y., Chen L., Zhao Y., Bai L., Hao X., Li X. (2020). Short-Chain Fatty Acids and Their Association with Signalling Pathways in Inflammation, Glucose and Lipid Metabolism. Int. J. Mol. Sci..

[17] Wang M., Wang B., Wang S., Lu H., Wu H., Ding M., Ying L., Mao Y., Li Y. (2021). Effect of Quercetin on Lipids Metabolism Through Modulating the Gut Microbial and AMPK/PPAR Signaling Pathway in Broilers. Front. Cell Dev. Biol..

[18] Hu Z., Li M., Cao Y., Akan O.D., Guo T., Luo F. (2022). Targeting AMPK Signaling by Dietary Polyphenols in Cancer Prevention. Mol. Nutr. Food Res..

[19] Angé M., Castanares-Zapatero D., De Poortere J., Dufeys C., Courtoy G.E., Bouzin C., Quarck R., Bertrand L., Beauloye C., Horman S. (2020). A1AMP-Activated Protein Kinase Protects against Lipopolysaccharide-Induced Endothelial Barrier Disruption via Junctional Reinforcement and Activation of the P38 MAPK/HSP27 Pathway. Int. J. Mol. Sci..

[20] Boyd R.A., Majumder S., Stiban J., Mavodza G., Straus A.J., Kempelingaiah S.K., Reddy V., Hannun Y.A., Obeid L.M., Senkal C.E. (2023). The Heat Shock Protein Hsp27 Controls Mitochondrial Function by Modulating Ceramide Generation. Cell Rep..

[21] Costea T., Hudiță A., Ciolac O.A., Gălățeanu B., Ginghină O., Costache M., Ganea C., Mocanu M.M. (2018). Chemoprevention of Colorectal Cancer by Dietary Compounds. Int. J. Mol. Sci..

[22] Wang K., Jin X., Chen Y., Song Z., Jiang X., Hu F., Conlon M.A., Topping D.L. (2016). Polyphenol-Rich Propolis Extracts Strengthen Intestinal Barrier Function by Activating AMPK and ERK Signaling. Nutrients.

[23] Ma H., Hu Y., Zhang B., Shao Z., Roura E., Wang S. (2022). Tea Polyphenol—Gut Microbiota Interactions: Hints on Improving the Metabolic Syndrome in a Multi-Element and Multi-Target Manner. Food Sci. Hum. Wellness.

[24] Xu W., Luo Y., Yin J., Huang M., Luo F. (2023). Targeting AMPK Signaling by Polyphenols: A Novel Strategy for Tackling Aging. Food Funct..

[25] Rini D.M., Yamamoto Y., Suzuki T. (2023). Partially Hydrolyzed Guar Gum Upregulates Heat Shock Protein 27 in Intestinal Caco-2 Cells and Mouse Intestine via MTOR and ERK Signaling. J. Sci. Food Agric..

[26] Rini D.M., Nakamichi Y., Morita T., Inoue H., Mizukami Y., Yamamoto Y., Suzuki T. (2024). Xylobiose Treatment Strengthens Intestinal Barrier Function by Regulating Claudin 2 and Heat Shock Protein 27 Expression in Human Caco-2 Cells. J. Sci. Food Agric..

[27] Angulo-López J.E., Flores-Gallegos A.C., Ascacio-Valdes J.A., Contreras Esquivel J.C., Torres-León C., Rúelas-Chácon X., Aguilar C.N. (2022). Antioxidant Dietary Fiber Sourced from Agroindustrial Byproducts and Its Applications. Foods.

[28] de Almeida Sousa Cruz M.A., de Barros Elias M., Calina D., Sharifi-Rad J., Teodoro A.J. (2024). Insights into Grape-Derived Health Benefits: A Comprehensive Overview. Food Prod. Process. Nutr..

[29] Pozuelo M.J., Agis-Torres A., Hervert-Hernández D., López-Oliva M.E., Muñoz-Martínez E., Rotger R., Goñi I. (2012). Grape Antioxidant Dietary Fiber Stimulates Lactobacillus Growth in Rat Cecum. J. Food Sci..

[30] Guarino M.P.L., Altomare A., Emerenziani S., Di Rosa C., Ribolsi M., Balestrieri P., Iovino P., Rocchi G., Cicala M. (2020). Mechanisms of Action of Prebiotics and Their Effects on Gastro-Intestinal Disorders in Adults. Nutrients.

[31] López-Oliva M.E., Agis-Torres A., Goñi I., Muñoz-Martínez E. (2010). Grape Antioxidant Dietary Fibre Reduced Apoptosis and Induced a Pro-Reducing Shift in the Glutathione Redox State of the Rat Proximal Colonic Mucosa. Br. J. Nutr..

[32] López-Oliva M.E., Pozuelo M.J., Rotger R., Muñoz-Martínez E., Goñi I. (2013). Grape Antioxidant Dietary Fibre Prevents Mitochondrial Apoptotic Pathways by Enhancing Bcl-2 and Bcl-XL Expression and Minimising Oxidative Stress in Rat Distal Colonic Mucosa. Br. J. Nutr..

[33] López-Oliva M.E., Agis-Torres A., García-Palencia P., Goñi I., Muñoz-Martínez E. (2006). Induction of Epithelial Hypoplasia in Rat Cecal and Distal Colonic Mucosa by Grape Antioxidant Dietary Fiber. Nutr. Res..

[34] Sánchez-Tena S., Lizárraga D., Miranda A., Vinardell M.P., García-García F., Dopazo J., Torres J.L., Saura-Calixto F., Capellà G., Cascante M. (2013). Grape Antioxidant Dietary Fiber Inhibits Intestinal Polyposis in ApcMin/+ Mice: Relation to Cell Cycle and Immune Response. Carcinogenesis.

[35] Derry M., Somasagara R., Raina K., Kumar S., Gomez J., Patel M., Agarwal R., Agarwal C. (2014). Target Identification of Grape Seed Extract in Colorectal Cancer Using Drug Affinity Responsive Target Stability (DARTS) Technique: Role of Endoplasmic Reticulum Stress Response Proteins. Curr. Cancer Drug Targets.

[36] Kaur M., Tyagi A., Singh R.P., Sclafani R.A., Agarwal R., Agarwal C. (2011). Grape Seed Extract Upregulates P21 (Cip1) through Redox-Mediated Activation of ERK1/2 and Posttranscriptional Regulation Leading to Cell Cycle Arrest in Colon Carcinoma HT29 Cells. Mol. Carcinog..

[37] Wang L., Zhan J., Huang W. (2020). Grape Seed Proanthocyanidins Induce Apoptosis and Cell Cycle Arrest of HepG2 Cells Accompanied by Induction of the MAPK Pathway and NAG-1. Antioxidants.

[38] Roy A.M., Baliga M.S., Elmets C.A., Katiyar S.K. (2005). Grape Seed Proanthocyanidins Induce Apoptosis through P53, Bax, and Caspase 3 Pathways. Neoplasia.

[39] Mantena S.K., Baliga M.S., Katiyar S.K. (2006). Grape Seed Proanthocyanidins Induce Apoptosis and Inhibit Metastasis of Highly Metastatic Breast Carcinoma Cells. Carcinogenesis.

[40] Saura-Calixto F., Goñi I. (2005). Functional Formulation Based on Antioxidant Dietary Fiber and Soluble Fiber. Patent.

[41] Pérez-Jiménez J., Serrano J., Tabernero M., Arranz S., Díaz-Rubio M.E., García-Diz L., Goñi I., Saura-Calixto F. (2008). Effects of Grape Antioxidant Dietary Fiber in Cardiovascular Disease Risk Factors. Nutrition.

[42] Touriño S., Pérez-Jiménez J., Mateos-Martín M.L., Fuguet E., Vinardell M.P., Cascante M., Torres J.L. (2011). Metabolites in Contact with the Rat Digestive Tract after Ingestion of a Phenolic-Rich Dietary Fiber Matrix. J. Agric. Food Chem..

[43] Touriño S., Fuguet E., Jáuregui O., Saura-Calixto F., Cascante M., Torres J.L. (2008). High-Resolution Liquid Chromatography/Electrospray Ionization Time-of-Flight Mass Spectrometry Combined with Liquid Chromatography/Electrospray Ionization Tandem Mass Spectrometry to Identify Polyphenols from Grape Antioxidant Dietary Fiber. Rapid Commun. Mass Spectrom..

[44] Pérez-Jiménez J., Sanz Y., Lamuela-Raventós R.M. (2025). (Poly) Phenols as Bioactive Constituents Linked to Dietary Fibre Metabolic Fate. Trends Endocrinol. Metab..

[45] Fernandes A., Mateus N., de Freitas V. (2023). Polyphenol-Dietary Fiber Conjugates from Fruits and Vegetables: Nature and Biological Fate in a Food and Nutrition Perspective. Foods.

[46] Hou C., Chen Y., Zhang W., Yu J., Ji M., Cai S., Guo W., Ji X., Sun L., Liu X. (2025). An Insight into the Full Aspects of Bound Polyphenols in Dietary Fiber: Interaction, Composition, Function and Foundation as Well as Alteration in Food Processing. Food Chem..

[47] Ding Y., Morozova K., Scampicchio M., Ferrentino G. (2020). Non-Extractable Polyphenols from Food By-Products: Current Knowledge on Recovery, Characterisation, and Potential Applications. Processes.

[48] Das T., Chatterjee N., Capanoglu E., Lorenzo J.M., Das A.K., Dhar P. (2023). The Synergistic Ramification of Insoluble Dietary Fiber and Associated Non-Extractable Polyphenols on Gut Microbial Population Escorting Alleviation of Lifestyle Diseases. Food Chem. X.

[49] Ramos-Romero S., Martínez-Maqueda D., Hereu M., Amézqueta S., Torres J.L., Pérez-Jiménez J. (2020). Modifications of Gut Microbiota after Grape Pomace Supplementation in Subjects at Cardiometabolic Risk: A Randomized Cross-Over Controlled Clinical Trial. Foods.

[50] Di Luccia B., Acampora V., Saggese A., Calabrò V., Vivo M., Angrisano T., Baccigalupi L., Ricca E., Pollice A. (2022). Modulation of Intestinal Epithelial Cell Proliferation and Apoptosis by Lactobacillus Gasseri SF1183. Sci. Rep..

[51] Whitman J.A., Doherty L.A., Pantoja-Feliciano de Goodfellow I.G., Racicot K., Anderson D.J., Kensil K., Karl J.P., Gibson G.R., Soares J.W. (2024). In Vitro Fermentation Shows Polyphenol and Fiber Blends Have an Additive Beneficial Effect on Gut Microbiota States. Nutrients.

[52] Maiuolo J., Bulotta R.M., Ruga S., Nucera S., Macrì R., Scarano F., Oppedisano F., Carresi C., Gliozzi M., Musolino V. (2024). The Postbiotic Properties of Butyrate in the Modulation of the Gut Microbiota: The Potential of Its Combination with Polyphenols and Dietary Fibers. Int. J. Mol. Sci..

[53] Cao X., Wang X., Ren Y., Sun Y., Yang Z., Ge J., Ping W. (2023). *Lonicera caerulea* L. Polyphenols Improve Short-Chain Fatty Acid Levels by Reshaping the Microbial Structure of Fermented Feces in Vitro. Front. Microbiol..

[54] Castrogiovanni C., Waterschoot B., De Backer O., Dumont P. (2018). Serine 392 Phosphorylation Modulates P53 Mitochondrial Translocation and Transcription-Independent Apoptosis. Cell Death Differ..

[55] Loughery J., Cox M., Smith L.M., Meek D.W. (2014). Critical Role for P53-Serine 15 Phosphorylation in Stimulating Transactivation at P53-Responsive Promoters. Nucleic Acids Res..

[56] Ramos S. (2008). Cancer Chemoprevention and Chemotherapy: Dietary Polyphenols and Signalling Pathways. Mol. Nutr. Food Res..

[57] Yi L., Wang Q., Luo H., Lei D., Tang Z., Lei S., Xiao H. (2022). Inhibitory Effects of Polyphenols-Rich Components From Three Edible Seaweeds on Inflammation and Colon Cancer in Vitro. Front. Nutr..

[58] Wu H., Li C., Cui M., Guo H., Chen S., Du J., Li H., Li Z. (2021). Polyphenols from Hippophae Rhamnoides Suppressed Colon Cancer Growth by Regulating MiRNA-Mediated Cell Cycle Arrest and Apoptosis in Vitro and in Vivo. J. Funct. Foods.

[59] Chen P., Zhang J.Y., Sha B.B., Ma Y.E., Hu T., Ma Y.C., Sun H., Shi J.X., Dong Z.M., Li P. (2017). Luteolin Inhibits Cell Proliferation and Induces Cell Apoptosis via Down-Regulation of Mitochondrial Membrane Potential in Esophageal Carcinoma Cells EC1 and KYSE450. Oncotarget.

[60] Randisi F., Perletti G., Marras E., Gariboldi M.B. (2025). Green Tea Components: In Vitro and In Vivo Evidence for Their Anticancer Potential in Colon Cancer. Cancers.

[61] Martín M.A., Goya L., Ramos S. (2016). Preventive Effects of Cocoa and Cocoa Antioxidants in Colon Cancer. Diseases.

[62] Vogler M., Braun Y., Smith V.M., Westhoff M.A., Pereira R.S., Pieper N.M., Anders M., Callens M., Vervliet T., Abbas M. (2025). The BCL2 Family: From Apoptosis Mechanisms to New Advances in Targeted Therapy. Signal Transduct. Target. Ther..

[63] Rath E., Haller D. (2022). Intestinal Epithelial Cell Metabolism at the Interface of Microbial Dysbiosis and Tissue Injury. Mucosal Immunol..

[64] Arike L., Seiman A., van der Post S., Rodriguez Piñeiro A.M., Ermund A., Schütte A., Bäckhed F., Johansson M.E.V., Hansson G.C. (2020). Protein Turnover in Epithelial Cells and Mucus along the Gastrointestinal Tract Is Coordinated by the Spatial Location and Microbiota. Cell Rep..

[65] Gu C., Fan X., Yu W., Gu C., Fan X., Yu W. (2023). Functional Diversity of Mammalian Small Heat Shock Proteins: A Review. Cells.

[66] Peng M., Yi W., Murong M., Peng N., Tong H., Jiang M., Jin D., Peng S., Liang W., Quan J. (2023). Akkermansia Muciniphila Improves Heat Stress-Impaired Intestinal Barrier Function by Modulating HSP27 in Caco-2 Cells. Microb. Pathog..

[67] Sun Q., Tian Q., Bravo Iniguez A., Sun X., Zhang H., Deavila J., Du M., Zhu M.J. (2024). AMPK Deficiency Increases DNA Methylation and Aggravates Colorectal Tumorigenesis in AOM/DSS Mice. Genes.

[68] Figueras T., Perdicaro D.J., Cacciamani V.E., Gil Lorenzo A.F., Suhaiman L., Antoniolli A., Vazquez Prieto M.A., Costantino V.V. (2025). Grape Pomace Extract, Rich in Phenolic Compounds, Attenuates Obesity-Induced Nephropathy by Modulating Energy Metabolism Dysregulation and Oxidative Stress in Mice Fed a High-Fat Diet. Food Funct..

[69] Saxton R.A., Sabatini D.M. (2017). MTOR Signaling in Growth, Metabolism, and Disease. Cell.

[70] Kaur H., Moreau R. (2021). MTORC1 Silencing during Intestinal Epithelial Caco-2 Cell Differentiation Is Mediated by the Activation of the AMPK/TSC2 Pathway. Biochem. Biophys. Res. Commun..

[71] Yibcharoenporn C., Muanprasat C., Moonwiriyakit A., Satitsri S., Pathomthongtaweechai N. (2025). AMPK in Intestinal Health and Disease: A Multifaceted Therapeutic Target for Metabolic and Inflammatory Disorders. Drug Des. Dev. Ther..

[72] Gasaly N., Hermoso M.A., Gotteland M. (2021). Butyrate and the Fine-Tuning of Colonic Homeostasis: Implication for Inflammatory Bowel Diseases. Int. J. Mol. Sci..

[73] Martínez-Ruiz M., Robeson M.S., Piccolo B.D. (2025). Fueling the Fire: Colonocyte Metabolism and Its Effect on the Colonic Epithelia. Crit. Rev. Food Sci. Nutr..

[74] Machado T.O.X., Portugal I., de Kodel H.A.C., Droppa-Almeida D., Dos Santos Lima M., Fathi F., Oliveira M.B.P.P., de Albuquerque-Júnior R.L.C., Dariva C., Souto E.B. (2024). Therapeutic Potential of Grape Pomace Extracts: A Review of Scientific Evidence. Food Biosci..

[75] Muscia Saez V., Perdicaro D.J., Cremonini E., Costantino V.V., Fontana A.R., Oteiza P.I., Vazquez Prieto M.A. (2025). Grape Pomace Extract Attenuates High Fat Diet-Induced Endotoxemia and Liver Steatosis in Mice. Food Funct..

[76] Zhou Y., Liu F. (2022). Coordination of the AMPK, Akt, MTOR, and P53 Pathways under Glucose Starvation. Int. J. Mol. Sci..

[77] Koo K.Y., Moon K., Song H.S., Lee M.S. (2025). Metabolic Regulation by P53: Implications for Cancer Therapy. Mol. Cells.

[78] Liang J., Shao S.H., Xu Z.X., Hennessy B., Ding Z., Larrea M., Kondo S., Dumont D.J., Gutterman J.U., Walker C.L. (2007). The Energy Sensing LKB1-AMPK Pathway Regulates P27(Kip1) Phosphorylation Mediating the Decision to Enter Autophagy or Apoptosis. Nat. Cell Biol..

[79] Dragoni S., Caridi B., Karatsai E., Burgoyne T., Sarker M.H., Turowski P. (2021). AMP-Activated Protein Kinase Is a Key Regulator of Acute Neurovascular Permeability. J. Cell Sci..

[80] Ochoa-Sanchez A., Sahare P., Pathak S., Banerjee A., Estevez M., Duttaroy A.K., Luna-Bárcenas G., Paul S. (2024). Evaluation of the Synergistic Effects of Curcumin-Resveratrol Co-Loaded Biogenic Silica on Colorectal Cancer Cells. Front. Pharmacol..

[81] Ming J., Chen J., Zheng F., Wang T., Du Y., Wang J., Shao X., Yang X., Wu C., Ye J. (2025). Dietary Quercetin Improves Growth Performance and Modulates Non-Specific Immunity, Antioxidant Capacity, and Lipid Metabolism via NF-ΚB, Nrf2, and AMPK Signaling Pathways in Black Carp (*Mylopharyngodon piceus*) Fed High-Fat Diets. Aquac. Rep..

[82] EFSA (2016). Scientific Opinion on Principles for Deriving and Applying Dietary Reference Values. EFSA J..

[83] Gavrieli Y., Sherman Y., Ben-Sasson S.A. (1992). Identification of Programmed Cell Death in Situ via Specific Labeling of Nuclear DNA Fragmentation. J. Cell Biol..

