# Supplementary material for: Sustainable Grape Antioxidant Dietary Fiber Preserves Proximal Colonic Homeostasis via Hsp27 and AMPK Signaling
Source: Int J Mol Sci. 2025 Oct 30;26(21):10564. doi: 10.3390/ijms262110564 (PMC12608008; doi:10.3390/ijms262110564)
Supplement: Supplementary file 1 [file ijms-26-10564-s001.zip › ijms-3933144-supplementary.pdf]

International Journal of
Molecular Sciences

MDPI

Table S1: Composition of the experimental diets (g/kg).

	Control diet	GADF diet
Casein	140	140
Cornstarch	465,69	465,69
Dyetrose	155	155
Sucrose	100	100
Cellulose	50	0
Mineral mix AIN-93M #210050	35	35
Vitamin mix AIN-93VX #310025	10	10
L-Cystine	1.80	1.80
Choline bitartrate	2.50	2.50
t- Butylhydroquinone	0,008	0,008
Soybean oil	40	40
Grape antioxidant dietary fibre (GADF)	0	50

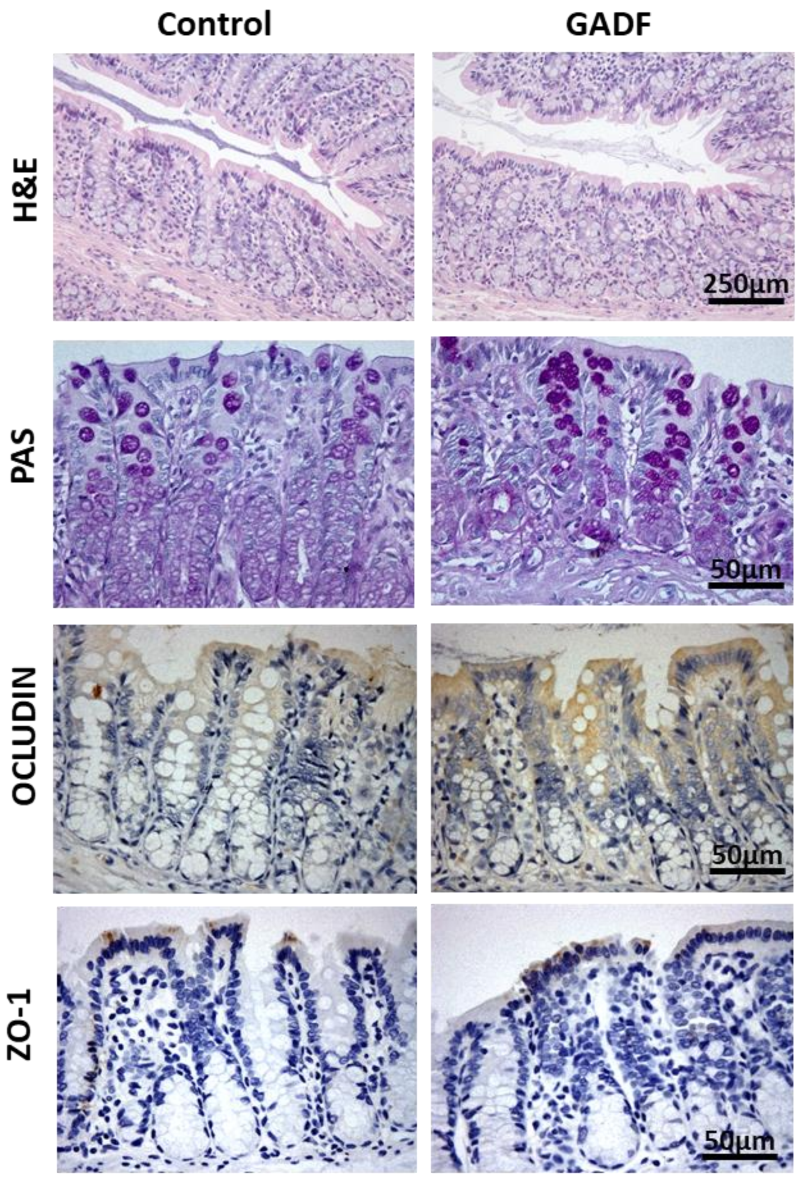

Figure S1. Effects of GADF on colonic morphology and barrier markers in rats. Representative histological and immunohistochemical images of proximal colon sections. Photomicrographs show H&E staining for tissue architecture and mucosal morphology, PAS staining for mucin goblet cells, and immunohistochemistry for barrier-related proteins occludin and ZO-1 in control and GADF-fed rats. Images correspond to representative samples included in the quantitative analyses presented in Table 1.
